# Radiotherapy and Immunotherapy for Head and Neck Cancer: Current Evidence and Challenges

**DOI:** 10.3389/fonc.2020.608772

**Published:** 2021-02-03

**Authors:** Jack M. Qian, Jonathan D. Schoenfeld

**Affiliations:** ^1^ Department of Radiation Oncology, Dana-Farber/Brigham and Women’s Cancer Center, Harvard Medical School, Boston, MA, United States; ^2^ Harvard Radiation Oncology Program, Massachusetts General Hospital/Brigham and Women’s Hospital/Dana-Farber Cancer Institute, Boston, MA, United States

**Keywords:** anti-PD-1, immunotherapy, radiation therapy, head and neck cancer, anti-PD-L1

## Abstract

Immune checkpoint inhibitors (ICI) have revolutionized cancer treatment over the past decade. However, although the immune landscape suggests a strong rationale for the use of these agents in patients with head and neck squamous cell carcinoma, the available clinical evidence indicates that most patients currently do not respond to ICI monotherapy. Radiotherapy is a primary treatment modality for many patients with locally advanced head and neck cancer. While ionizing radiation traditionally has been thought to act in a purely cytotoxic fashion, a growing body of preclinical studies have demonstrated additional profound immunomodulatory effects. Consequently, there has been a surge of interest in the potential synergy between radiotherapy and immunotherapy, both the potential for radiotherapy to augment the systemic anti-tumor immune response and the potential for immunotherapy to improve in-field tumor response to radiation. In this review, we summarize the current preclinical and clinical evidence for radioimmunotherapy, with a particular focus on studies directly relevant to head and neck squamous cell carcinoma, as well as existing challenges and future directions for this emerging field.

## Introduction

Head and neck cancers comprise a significant portion of the global cancer burden; when aggregating subsites, they are the 8^th^ most common cancer worldwide by both incidence and mortality (1). Although the vast majority of head and neck cancers are squamous cell carcinomas (HNSCC) and have traditionally been associated with tobacco and alcohol use, HPV-associated oropharyngeal squamous cell carcinoma (SCC) has emerged as a new disease entity with markedly different biological behavior (2).

Ever since the foundational work of Henri Coutard, who was the first to use X-rays to treat laryngeal cancer almost 100 years ago (3), radiation therapy has played a key role in the treatment of HNSCC. Radiation continues to be used extensively both in the curative as well as palliative setting, although the distinction between the two is now sometimes blurred with growing recognition of the oligometastatic state, where patients with limited numbers of metastases can achieve prolonged survival, or even cure (4, 5). Technological advancements, both in imaging as well as treatment delivery, have enabled more precise radiation treatment that has reduced treatment-related morbidity and improved patient outcomes. However, even with the use of modern radiation techniques, there are still opportunities for further improvement (4).

The immune system has a critical role in tumor development, and the development of immune evasion by tumors is a key step in carcinogenesis (6, 7). Attempts to reinvigorate an anti-tumor immune response have been widely integrated into practice following the development of the immune checkpoint inhibitors (ICIs) targeted against the immune checkpoint receptors cytotoxic T-lymphocyte-associated protein 4 (CTLA-4), programmed cell death protein 1 (PD-1), and programmed death-ligand 1 (PD-L1). Since the initial FDA approval of ipilimumab (a CTLA-4 inhibitor) in 2011 for the treatment of metastatic melanoma based on a proven overall survival advantage (8), antibodies blocking CTLA-4 and PD-1/PD-L1 have been tested and approved across a wide spectrum of malignancies. In HNSCC, both pembrolizumab and nivolumab (PD-1 inhibitors) have gained FDA approval for use in recurrent/metastatic HNSCC after progression through platinum-based chemotherapy (9–11). Pembrolizumab additionally has been approved in the US for use in the first line setting in patients with recurrent/metastatic HNSCC, either in combination with chemotherapy or alone as monotherapy depending on tumor/tumor microenvironment PD-L1 expression (12).

Unfortunately, overall response rates to PD-1 inhibitors in unselected patients with HNSCC remain low at approximately 10–20% (9–12), although patients who do respond can have long-lasting, durable remissions, as has been the case with other solid tumor patients who respond to PD-1 blockade (13). The possibility of durable long-term response has been a driver of the rapid uptake in clinical practice and has invigorated efforts to develop predictive biomarkers. Tumor mutational burden, a potential surrogate for tumor neoantigens that can be recognized by the immune system, is one such biomarker, leading to the first ever histology-agnostic FDA approval of the PD-1 inhibitor pembrolizumab for mismatch repair deficient tumors of any histology (14, 15), though there is increasing recognition that the types and functional nature of mutations may be as important as the number of mutations present (16). PD-L1 expression on both tumor cells and infiltrated immune cells has also been explored as a biomarker across several histologies with varying results; in HNSCC, subgroup analyses of Checkmate 141, KEYNOTE-040, and KEYNOTE-048 all suggest that higher PD-L1 expression does correlate with the likelihood of survival benefit (10–12). It is less clear whether patients with low or no PD-L1 expression still benefit from PD-1 directed therapy; analyses of Checkmate 141 and KEYNOTE-048 show questionable benefit for the PD-L1 negative subgroup when comparing the treatment and control arms (11, 17). Finally, for HNSCC patients, HPV-associated malignancies with relatively fewer tumor mutations as compared to tobacco-associated malignancies may also respond to immune checkpoint blockade as novel viral-associated neoantigens might be recognized by the immune system. Indeed, subgroup analyses of the Checkmate 141 and KEYNOTE-040 trials did not show any clear differences in response or clinical benefit based on p16 expression status (a surrogate for HPV-associated tumors) (10, 11).

**Figure 1:**
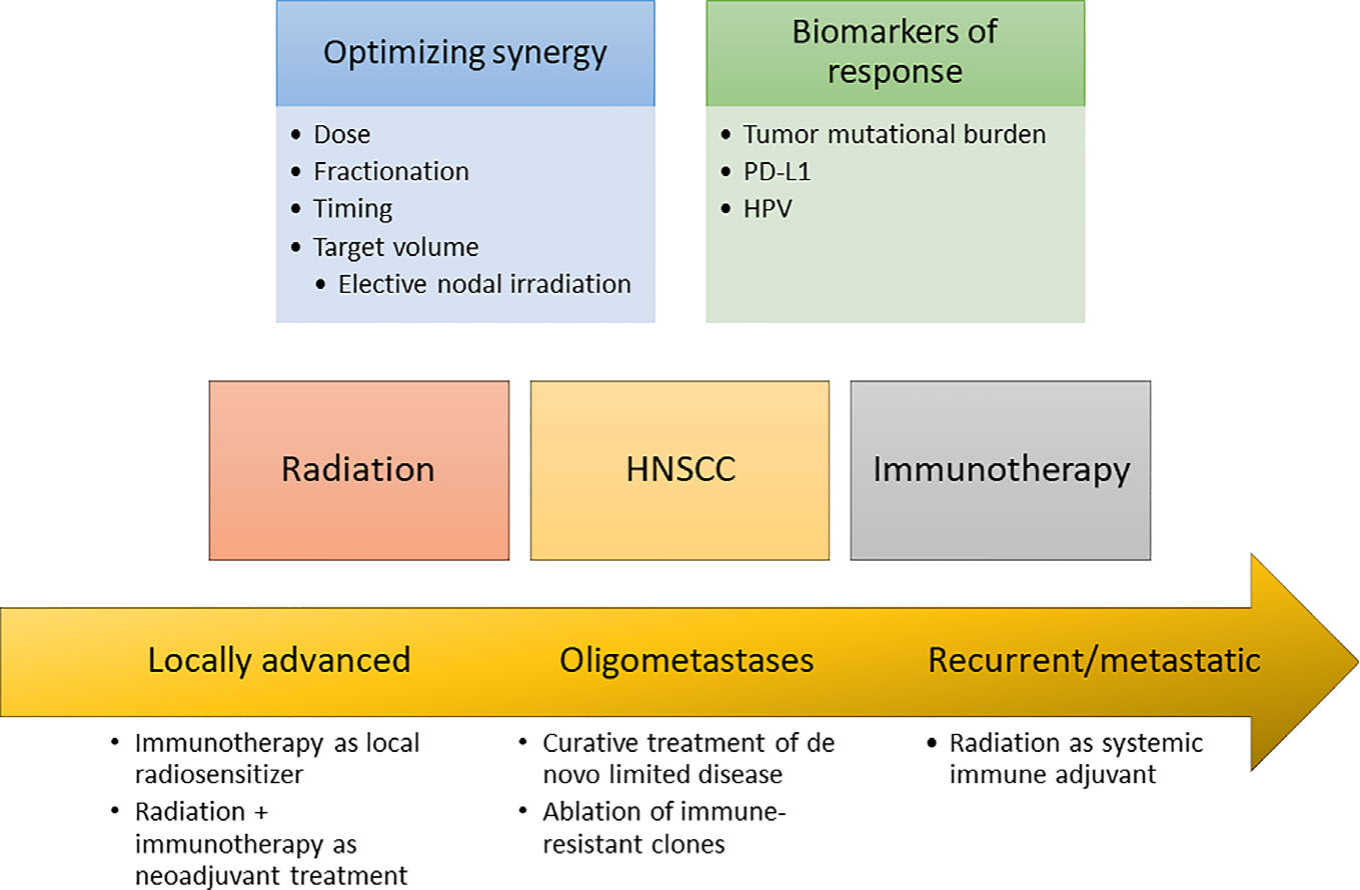
Opportunities for radioimmunotherapy in HNSCC.

In addition to better patient selection through the use of predictive biomarkers, augmenting the anti-tumor immune response with other therapies could also improve immunotherapy response rates. Radiation therapy increasingly has been recognized to have diverse immunomodulatory effects, and there has consequently been intense interest in possible synergism between radiation therapy and immunotherapy. In this review, we will summarize the preclinical data that illustrate the immune effects of radiation therapy, review the unique immune landscape of HNSCC, and finally discuss both current preclinical and clinical data relevant to the combination of radiation therapy and immunotherapy specifically in HNSCC (**Figure 1**).

## Immune Effects of Radiation Therapy

Traditionally, the anti-tumor effects of radiation therapy have been attributed to direct cytotoxicity secondary to the induction of DNA damage, and while it was known over 40 years ago that radiation therapy also depends on an intact immune system to exert its full anti-tumor effect (18), the interaction between the immune system and radiation therapy has garnered more interest in the past two decades. It is now recognized that the immune effects of radiation may contribute significantly to an anti-tumor response; however, these immune effects are also quite complex and can be both immunostimulatory and immunosuppressive.

Radiation can induce immunogenic cell death, which gives rise to adaptive immune responses (19, 20). Many mechanisms can be involved in this process, and a full detailed review is beyond the scope of this discussion. However, recent studies have shown radiation can promote release of danger-associated molecular patterns such as calreticulin, ATP, and HMGB (20, 21). Radiation also induces release of cytosolic DNA, which triggers the cGAS/STING pathway to upregulate production of type-I interferon (22, 23). Type-I interferon is crucial for the activation of dendritic cells, which ultimately recruit and prime T-cells. These signals together are critical for the initial development of an immune response specific to tumor neoantigens.

Radiation can promote anti-tumor immunity through additional mechanisms. Radiation can diversify antigen presentation by tumor cells through promotion of intracellular peptide degradation as well as upregulation of MHC expression (24, 25). This ultimately can enhance recognition and tumor cell killing by cytotoxic T-cells (26). Radiation has also been associated with increased production of other immune stimulating cytokines and chemokines, which together can promote the infiltration of T-cells into tumors and modulate the function of these T-cells, as well as dendritic cells and macrophages (21).

Radiation also has immunosuppressive effects that could be detrimental to an anti-tumor immune response. Lymphocytes are radiosensitive, with *in vitro* studies demonstrating that 3 Gy of radiation is enough to deplete 90% of human lymphocytes (27). This may be overly simplistic, however, as more recent work suggests differential radiosensitivity of T-cell subtypes. Pre-existing intra-tumoral T-cells in particular appear to be potentially more radioresistant than either circulating T-cells or lymphoid tissue T-cells. These intra-tumoral T cells survive even high doses (20 Gy) of radiation in preclinical studies and can develop a similar transcriptomic profile to tissue-resident memory T-cells, which are also thought to be radioresistant (28, 29). These intra-tumoral T-cells can mediate some of the anti-tumor immune effects of high dose radiation. Regardless, clinical data suggest that radiation-induced lymphopenia may be a negative prognostic factor in patients treated with PD-1 and CTLA-4 inhibitors (30).

Within the local tumor microenvironment, a variety of inhibitory immune cells, such as T-regulatory cells (Tregs), myeloid-derived suppressor cells (MDSCs), and tumor-associated macrophages (TAMs, and specifically M2 macrophages), are often already present. In several studies, radiation increases recruitment of these inhibitory immune cells and can also modulate their function towards an even more immunosuppressive phenotype (21). There may also be dose-dependent effects of radiation; for instance, Vanpouille-Box et al. demonstrated that as radiation doses were escalated to 12–18 Gy, there was induction of Trex1, a DNA exonuclease which degrades cytosolic DNA and thus prevents activation of the cGAS/STING pathway (23). The balance between competing activating and inhibitory immune responses, then, likely plays a key role in the probability of a successful anti-tumor immune response and provides opportunity for therapeutic intervention.

## Immune Landscape of HNSCC

Work over the past decade has helped characterize the immune landscape of HNSCC. As noted above, HPV-associated oropharyngeal SCC is a distinct disease entity from other non-HPV-driven, tobacco-associated HNSCC, with a distinct immune profile. Using data from The Cancer Genome Atlas, Mandal et al. showed that HPV-positive tumors were significantly more immune infiltrated than HPV-negative tumors (31). However, both HPV-positive and HPV-negative HNSCC had the highest rate of immunosuppressive Treg infiltration among 10 different cancer types. There was a correlation between the molecular smoking signature of HNSCC tumors and increased tumor mutational burden, but also conversely an inverse association between the molecular smoking signature and immune infiltration, despite this higher tumor mutation burden (and therefore presumably increased neoantigen load). This suggests that tobacco-associated tumors can still be immunologically cold despite their higher mutational load. Further work has demonstrated that HPV-positive tumors are associated with increased T-cell receptor diversity, higher levels of immune cytolytic activity, and an overall enriched inflammatory response (32, 33). The anatomic subsite where head and neck cancer develops likely plays a key role in tumor immunity as well; the oropharynx contains particularly lymphoid-rich tissue, and this unique immune environment may explain why the improved prognosis for HPV-driven HNSCC is largely limited to oropharyngeal tumors (34). Additional work on oropharyngeal SCC has confirmed a higher degree of infiltration of CD8+ T-cells in HPV-positive *vs* HPV-negative tumors (35). Overall, these studies suggest that the increased sensitivity of HPV-associated oropharyngeal SCC to chemotherapy and radiation therapy may at least in part be mediated through immune mechanisms (36, 37), and that differing immunotherapeutic approaches may be optimal for HPV-positive and HPV-negative HNSCC.

HNSCC also appears to be uniquely associated with high levels of natural killer (NK) cell infiltration, even when compared to other highly-immune infiltrated cancer types (31, 35). Patients with high levels of NK cell infiltration were also found to have improved survival compared to those with low levels of infiltration (31). The potential anti-tumor effects of NK cells is an emerging area of research and has been reviewed elsewhere (38); currently, there is limited clinical data on their role in HNSCC, or whether opportunities for synergy between NK-directed therapies and radiation exist.

## Preclinical Evidence for Radioimmunotherapy in HNSCC Models

### Augmenting Anti-tumor Cellular Immunity

Preclinical work in HNSCC models has demonstrated synergy between radiation and immunotherapy. In a poorly immunogenic orthotopic HNSCC mouse model, Oweida et al. demonstrated effective tumor cell killing when both 10 Gy of radiation and an anti PD-L1 antibody were administered together, but not for either treatment individually (39). Tumor control was correlated with increased tumor T-cell infiltration and was abrogated when CD4+ and CD8+ T-cells were depleted. In addition, although much of research on anti-tumor immunity has focused on the role of T-cells, work from Kim et al. in a mouse model of HPV-associated HNSCC suggests that the combination of radiation and PD-1 inhibition also promotes maturation and activation of B-cells, leading to the development of memory B-cells, plasma cells, and antigen-specific B-cells, as well as increasing formation of B-cell germinal centers in tumor draining lymph nodes (40). Finally, there is growing interest in harnessing additional molecular pathways to promote anti-tumor immunity. For instance, in a mouse model of HPV-driven carcinoma, Dillon et al. demonstrated that inhibitors of ATR, a key protein in the DNA damage response pathway, significantly sensitized tumors to radiation, and this effect was correlated with upregulation of interferon-stimulated genes and a significant increase in innate immune cell infiltration into the tumor microenvironment (41). Xiao et al. showed that ASTX600, an inhibitor of IAP1/2 and XIAP, proteins that modulate apoptosis and the tumor necrosis factor signaling pathway, significantly enhanced T-cell mediated tumor cell killing when combined with radiation and PD-1 inhibition in a mouse model of oral cavity carcinoma (42).

### Decreasing an Immunosuppressive Microenvironment

The immunosuppressive microenvironment remains a challenge even with combined radiation and immunotherapy. In a follow-up study, Oweida et al. demonstrated that the anti-tumor immune responses to combined radiation and PD-1 inhibition in their HNSCC mouse model were ultimately transient, as compensatory mechanisms of immune evasion were activated, including upregulation of another immune checkpoint, TIM-3, as well as increased tumor infiltration of Tregs (39, 43). Adding an anti-TIM-3 antibody further delayed tumor growth, but the response was still not durable; only targeted depletion of Tregs was able to induce durable immunologic memory. Another group has explored the use of cyclophosphamide and an inhibitor of inducible nitric oxide synthase (iNOS) as immunomodulatory agents in a mouse model of HPV-associated HNSCC. When combined with traditional chemoradiation, addition of these two agents increased the CD8+ T-cell/Treg ratio and decreased immunosuppression (44). In this particular model system the combination of radiation with PD-1 and CTLA-4 inhibition only minimally altered the immunologically cold tumor microenvironment, but the addition of cyclophosphamide and the iNOS inhibitor shifted the balance of infiltrated immune cells away from immunosuppressive types (such as MDSCs) to those more associated with anti-tumor immunity (such as dendritic cells and anti-tumor M1 macrophages). This led to an increased CD8+ T-cell-dependent response and complete tumor rejection in more than 70% of the treated mice (45). This is now being investigated in a clinical trial, NCT03844763, which explores the use of cyclophosphamide, avelumab (a PD-L1 inhibitor), and radiation therapy in the treatment of recurrent/metastatic HNSCC.

### Radiation Dose and Fractionation Effects

Additional studies have demonstrated the importance of radiation dose and fractionation in generating an effective anti-tumor immune response. Consistent with work in other diseases (46), Morisada et al. showed in a syngeneic mouse oral cavity carcinoma model that hypofractionated radiation (16 Gy in two fractions) was associated with preservation of both peripheral and tumor-infiltrating lymphocytes, reduction of both peripheral and tumor-associated MDSCs, and increased expression of interferon genes, when compared to conventionally fractionated radiation (20 Gy in 10 fractions) (47). Moreover, analysis of the draining lymph nodes (which notably were included within the radiation fields) suggested that 20 Gy in 10 fractions suppressed local tumor-specific T-cell responses. Consequently, only 16 Gy in two fractions demonstrated synergy with an anti-PD-1 antibody in these mice. Additional work by this group suggests a dose-dependent effect of radiation on both antigen release and T-cell priming, with 8 Gy in a single fraction enhancing these pathways compared to 2 Gy in a single fraction, resulting in increased tumor cell susceptibility to T-cell mediated killing (48). However, the doses used in these preclinical models differ from those used in clinical practice, as do the size of the treated tumors, and so it is uncertain how these findings might translate to the treatment of patients.

## Clinical Evidence for Radioimmunotherapy in HNSCC

### Recurrent/Metastatic Setting

Despite the widespread use of ICIs in advanced malignancies, prospective clinical data on their combination with radiation therapy remain scarce, particularly in HNSCC. The unique immune-related adverse effects (irAEs) that have been observed with ICIs are now well established (49) and there have been concerns that the pro-inflammatory effects of radiation could enhance toxicities when combined with ICIs. Reassuringly, however, most of the available clinical data to date suggests that the combination of radiation and ICIs is generally well tolerated (50). For instance, in a cohort of 133 patients with metastatic melanoma, non-small cell lung cancer (NSCLC), or renal cell cancer who received palliative radiation to a wide range of anatomic sites, Bang et al. demonstrated numerically higher rates of irAEs when radiation was given within 14 days of immunotherapy, but the toxicities were generally mild with rates of grade 3+ toxicity less than 10% (51). Similarly, a prospective phase I trial of pembrolizumab and stereotactic body radiotherapy (SBRT) in patients with a variety of metastatic solid tumors also demonstrated a grade 3+ toxicity rate of less than 10% (52). Notably, this study did include four patients with HNSCC, and radiation was delivered to two distinct anatomic sites in more than 60% of the cohort. Finally, a phase 2 trial which randomized 62 patients with metastatic HNSCC to nivolumab with or without SBRT to a single metastatic site did not find a significant difference in either grade 3–5 adverse events (13% for nivolumab alone *vs* 10% for nivolumab with SBRT, p = 0.70) or any grade adverse events (70% for nivolumab alone *vs* 87% for nivolumab with SBRT, p = 0.12) with the addition of SBRT (53).

Nevertheless, a few key issues must be considered when interpreting these and other safety data. Just as dose and fractionation likely affect potential anti-tumor immunity induced by radiation (as demonstrated in preclinical work), it is probable that these parameters influence potential toxicities when combined with ICIs. The relative timing of radiation and immunotherapy is likely to be important as well; notably, radiation recall, a relatively rare, unpredictable, and poorly understood phenomenon wherein an inflammatory reaction can develop in previously irradiated tissue following administration of a new systemic agent (54), has now been reported following ICI administration (55, 56). Additionally, the anatomic site treated with radiation could influence the side effect profile of combination treatment; for instance, the landmark PACIFIC trial, which demonstrated a significant overall survival benefit to adjuvant durvalumab (an anti-PD-L1 antibody) after definitive chemoradiation for stage III NSCLC, also showed an increase in any-grade pneumonitis with the addition of durvalumab (although rates of clinically relevant pneumonitis, i.e. grade 3+, were similar between treatment groups and low overall) (57). Within the brain, there is a potential increased risk of developing radiation necrosis after treatment of brain metastases with combined ICIs and radiation (58, 59). Finally, as discussed earlier, in certain settings radiation can induce lymphopenia, which could ultimately interfere with the efficacy of ICIs (30). These data highlight the importance of collecting robust radiation treatment and toxicity data to facilitate future analyses as we study combination radiation and immunotherapy treatments.

There are very few efficacy data relevant to the addition of radiation to ICIs in patients with recurrent or metastatic HNSCC. In general, the primary rationale for radiation in this setting is to help stimulate a systemic anti-tumor immune response, or abscopal effect. This is particularly difficult to study retrospectively, as disentangling a true abscopal effect from a delayed response to immunotherapy is challenging (60). The only available prospective data for HNSCC comes from the randomized phase 2 trial noted above, in which 62 patients with metastatic HNSCC were randomized to nivolumab with or without SBRT to a single metastatic site (9 Gy ×3 fractions, between the first and second doses of nivolumab). Ultimately, there was no improvement in overall response rate (34.5% for nivolumab alone *vs* 29.0% for nivolumab with SBRT, p = 0.86) (53). In NSCLC, a similarly designed phase 2 trial of pembrolizumab with or without SBRT to a single metastatic site in patients with advanced NSCLC also failed to meet its primary endpoint, although it did demonstrate a doubling of overall response rate with the addition of SBRT that was not statistically significant (18% for pembrolizumab alone *vs* 36% for pembrolizumab with SBRT, p = 0.07) (61). Differences between the designs of these two studies include the anti-PD-1 agent used (nivolumab *vs* pembrolizumab), the type of cancer (HNSCC *vs* NSCLC), timing of SBRT (between first and second dose of nivolumab *vs* prior to starting pembrolizumab), and dose of SBRT (9 Gy ×3 fractions *vs* 8 Gy ×3 fractions). Given the results of these trials, further research is clearly needed; **Table 1** summarizes ongoing trials that will help address these questions specifically in patients with recurrent/metastatic HNSCC. Notably, however, only a few of these studies are randomized, and so any efficacy data will require confirmation in larger, phase 3 trials.

**Table 1:** Ongoing trials evaluating combinations of ICIs and radiation in the management of recurrent/metastatic HNSCC.

NCT#	Title	Inclusion criteria	Treatment arms	Timing	Phase
NCT03539198	Study of Proton SBRT and Immunotherapy for Recurrent/Progressive Locoregional or Metastatic Head and Neck Cancer	Recurrent/metastatic HNSCC, ≥2 metastatic sites	1: nivolumab given every 2 weeks, with proton SBRT to one metastatic site administered with cycle 3	concurrent	n/a
NCT03283605	Immunotherapy and SBRT for Metastatic Head and Neck Carcinomas	Metastatic HNSCC, ≥2 metastatic sites	1: durvalumab + tremelimumab for four cycles (4 weeks each), SBRT between cycles 2 and 3	concurrent	1/2
NCT03844763	CONFRONT: Targeting the Tumor Microenvironment in R/M SCCHN	Recurrent/metastatic HNSCC	1: avelumab, cyclophosphamide, and radiation (8 Gy/1 fx) to a single site 1 week after first dose of avelumab	concurrent	1/2
NCT03522584	Durvalumab, Tremelimumab and Hypofractionated Radiation Therapy in Treating Patients With Recurrent or Metastatic Head and Neck Squamous Cell Carcinoma	Recurrent/metastatic HNSCC; progression through prior PD-1/PD-L1 inhibitor	1: durvalumab + tremelimumab for four cycles (4 weeks each) followed by durvalumab alone for nine cycles; SBRT during week 3 in three fractions, every other day	concurrent	1/2
NCT03474497	UCDCC#272: IL-2, Radiotherapy, and Pembrolizumab in Patients Refractory to Checkpoint Blockade	Recurrent/metastatic HNSCC; progression through prior PD-1/PD-L1 inhibitor	1: one cycle of pembrolizumab, then SBRT (24 Gy/3 fx) and intratumoral injection of interleukin-2 during cycle 2, then additional pembrolizumab	concurrent	1/2
NCT03317327	REPORT: REirradiation and Programmed Cell Death Protein 1 (PD-1) Blockade on Recurrent Squamous Cell Head and Neck Tumors	Recurrent HNSCC after prior radiation or second primary HNSCC	1: nivolumab with re-irradiation to 60 Gy (in 1.5 Gy bid fx), followed by nivolumab for up to 12 months	concurrent	1/2
NCT04340258	Trial Combining Pembrolizumab and Cesium 131 Brachytherapy With Salvage Surgery in HNSCC	Resectable recurrent HNSCC after prior surgery or radiation	1: one dose of pembrolizumab, then salvage surgery with implantation of Cesium-131 brachytherapy seeds (60–70 Gy), followed by adjuvant pembrolizumab for 6 months	concurrent	1/2
NCT04454489	Quad Shot Radiotherapy in Combination With Immune Checkpoint Inhibition	Recurrent/metastatic HNSCC	1: pembrolizumab given every 3 weeks; quad-shot radiation (14.8 Gy in 4 bid fx) between cycles 2 and 3	concurrent	2
NCT03313804	Priming Immunotherapy in Advanced Disease With Radiation	Recurrent/metastatic HNSCC	1: nivolumab, pembrolizumab, or atezolimuab, with either SBRT (BED > 100 Gy) or 30 Gy fractionated RT	concurrent	2
NCT03386357	Radiotherapy With Pembrolizumab in Metastatic HNSCC	Recurrent/metastatic HNSCC, ≥2 metastatic sites, progression through platinum-based therapy	1: radiation to 1–3 metastases (36 Gy/12 fx), with pembrolizumab starting between fraction 3 and 4	concurrent	2
			2: pembrolizumab alone		
NCT03511391	CHEERS: CHEckpoint Inhibition in Combination With an Immunoboost of External Body Radiotherapy in Solid Tumors	Recurrent/metastatic HNSCC, progression through platinum-based therapy	1: 2 cycles of nivolumab, then SBRT to 1–3 metastases (24 Gy/3 fx) prior to cycle 3	concurrent	2
			2: nivolumab alone		
NCT03085719	Targeting PD-1 Therapy Resistance With Focused High or High and Low Dose Radiation in SCCHN	Metastatic HNSCC, progression through prior PD-1 inhibition, ≥3 metastatic sites	1: pembrolizumab and high dose SBRT (3 fx) to one metastatic site	concurrent	2
			2: pembrolizumab and high dose SBRT (3 fx) to one metastatic site, and low dose radiation (2 fx) to another site		
NCT03546582	KEYSTROKE: SBRT +/− Pembrolizumab in Patients With Local-Regionally Recurrent or Second Primary Head and Neck Carcinoma	Recurrent HNSCC after prior radiation or second primary HNSCC	1: reirradiation with SBRT over 2 weeks, then pembrolizumab every 3 weeks for up to 2 years	sequential	2
			2: reirradiation with SBRT over 2 weeks		
NCT03521570	Intensity-Modulated Radiation Therapy & Nivolumab for Recurrent or Second Primary Head & Neck Squamous Cell Cancer	Recurrent HNSCC after prior radiation or second primary HNSCC	1: one dose of nivolumab, then radiation with concurrent nivolumab, then adjuvant nivolumab for 5 months	concurrent + sequential	2
NCT02289209	Reirradiation With Pembrolizumab in Locoregional Inoperable Recurrence or Second Primary Squamous Cell CA of the Head and Neck	Unresectable recurrent HNSCC after prior radiation or second primary HNSCC	1: pembrolizumab with re-irradiation to 60 Gy (in 1.2 Gy bid fx), followed by pembrolizumab for 3 months	concurrent + sequential	2
NCT02684253	Screening Trial of Nivolumab With Image Guided, Stereotactic Body Radiotherapy (SBRT) Versus Nivolumab Alone in Patients With Metastatic Head and Neck Squamous Cell Carcinoma (HNSCC)	Metastatic HNSCC, ≥2 metastatic sites	1: one cycle of nivolumab, then SBRT (27 Gy/3 fx) with the 2nd cycle, followed by additional nivolumab	concurrent	2
			2: nivolumab alone		

BED, biologically effective dose; bid, twice a day; fx, fraction; HNSCC, head and neck squamous cell carcinoma; ICIs, immune checkpoint inhibitors; SBRT, stereotactic body radiotherapy.

Finally, as noted above, there is growing recognition of an oligometastatic disease state. Contrary to previous conceptualization of metastatic disease as inevitably widespread and thus incurable, the oligometastatic hypothesis suggests that there is a wide range of metastatic potential that varies among different cancers and from patient to patient, and that an intermediate state likely exists between purely localized disease and widely metastatic disease, wherein a limited number of metastases might develop with limited further metastatic potential (62). Aggressive local treatment of patients with limited metastases would thus potentially offer a significant survival benefit. Results from several randomized phase 2 trials have supported this hypothesis (though notably HNSCC was not represented in any of these studies) (63–67). Consequently, there is interest in the addition of ICIs to radiation in this population of patients to improve outcomes (68). In this setting, radiation would be administered at ablative doses to all metastatic sites, and so the addition of ICIs would also be intended to augment the local effects of radiation at each treatment site. To our knowledge, no prospective clinical data has yet been published on the combination of radiation and ICIs in patients with oligometastatic HNSCC, though there is at least one ongoing clinical trial (NCT03283605, which examines the use of durvalumab, tremelimumab [a CTLA-4 inhibitor], and SBRT in patients with HNSCC with fewer than 10 metastases).

Related to the overall concept of oligometastases is oligoprogression, or the development of a limited number of progressive metastatic lesions after a period of stability on systemic therapy (69). In the context of ICIs, oligoprogression may herald general immune escape in patients who had previously been responding to treatment. However, in certain cases oligoprogression may develop as the result of resistant tumor clones that lack particular tumor antigens or antigen presentation, or because of differences in the underlying immune microenvironment of the anatomic site that permit localized immune escape (e.g. brain) (70, 71). If this is the case, local treatment such as radiation to these oligoprogressive sites may enable the patient to continue to derive benefit from ICIs (72–74). This paradigm is being tested prospectively in SCCHN (NCT03085719).

### Locally Advanced/Definitive Setting

ICIs are being investigated in the setting of curative treatment of earlier stages of disease across all cancer types, including HNSCC. Addition of ICIs to radiation in this setting would be intended to potentially augment the local effects of radiation (i.e. as a radiosensitizer) and address micrometastatic disease. Several possible combinations are under investigation—immunotherapy added to a chemoradiation regimen to intensify therapy (for patients with currently poor outcomes), immunotherapy given concurrently with radiation instead of chemotherapy or with a lower dose of radiation (potentially as a way to reduce treatment morbidity while maintaining overall efficacy), or immunotherapy administered adjuvantly and/or as induction (i.e. sequential therapy). To date adjuvant immunotherapy has proven successful in NSCLC; as noted earlier, the PACIFIC trial demonstrated a significant and meaningful overall survival benefit for adjuvant durvalumab starting within 6 weeks of completing standard chemoradiation for unresectable stage III NSCLC, with an increase in 2-year overall survival from 55.6 to 66.3% (75). Of note, the magnitude of benefit was greater patients who were randomized within 2 weeks of completing chemoradiation. Adjuvant immunotherapy also has newly demonstrated success in esophagogastric cancer; Checkmate-577 demonstrated improved disease-free survival with the administration of adjuvant nivolumab following neoadjuvant chemoradiation and surgical resection in patients with esophageal and gastroesophageal cancer, though full trial results have yet to be presented (76).

As shown in **Table 2**, ongoing trials are evaluating various combinations of radiation and ICIs for HNSCC in the definitive setting, and several have now reported safety data. In general, combinations of PD-1/PD-L1 inhibitors with definitive radiation appear well tolerated with no unexpected toxicities. KEYCHAIN is a randomized phase 2 study of radiation combined with concurrent and adjuvant pembrolizumab compared with radiation and concurrent cisplatin in intermediate-risk p16-positive HNSCC; the safety lead-in phase of the study found only one dose-limiting toxicity (grade 4 adrenal insufficiency) among eight patients in the pembrolizumab arm, and so the trial has proceeded to its phase 2 component (77). A single arm phase 2 trial of radiation administered with concurrent and adjuvant pembrolizumab in cisplatin-ineligible patients with locally advanced HNSCC similarly demonstrated relatively low toxicity in the first 12 enrolled patients, and 11 of 12 patients received all planned cycles of pembrolizumab (78). Finally, PembroRad is a randomized phase 2 trial of radiation combined with concurrent pembrolizumab *versus* radiation combined with concurrent cetuximab, again in cisplatin-ineligible patients with locally advanced HNSCC. There have been 133 patients randomized in a 1:1 fashion, and the pembrolizumab arm was found to have significantly less mucositis or dermatitis within the radiation field than the cetuximab arm (79).

**Table 2:** Ongoing trials evaluating combinations of ICIs and radiation in the definitive management of locally advanced HNSCC.

NCT#	Title	Inclusion criteria	Treatment arms	Timing	Phase
NCT02819752	PEmbrolizumab Combined With Chemoradiotherapy in Squamous Cell Carcinoma of the Head and Neck (PEACH)	LA HNSCC	1: pembrolizumab added to standard chemoradiation, three doses concurrently, four doses adjuvantly	concurrent + sequential	1
NCT04477759	Dose-Escalated Hypofractionated Adaptive Radiotherapy for Head and Neck Cancer (DEHART)	LA HNSCC, cisplatin-ineligible, or primary metastatic HNSCC	1: MR-guided hypofractionated radiation (50–60 Gy/15 fx); atezolizumab given with fraction 1 and 11 of radiation, then every 4 weeks for up to 1 year	concurrent + sequential	1
NCT03509012	CLOVER: Immunotherapy in Combination With Chemoradiation in Patients With Advanced Solid Tumors	LA HNSCC	1: durvalumab concurrent with standard radiation and cisplatin	concurrent	1
NCT02764593	RTOG 3504: Safety Testing of Adding Nivolumab to Chemotherapy in Patients With Intermediate and High-Risk Local-Regionally Advanced Head and Neck Cancer	LA HNSCC, intermediate or high risk	1: one dose of nivolumab as induction, then radiation (70 Gy/35 fx) and nivolumab with weekly cisplatin, then adjuvant nivolumab for seven doses	concurrent + sequential	1
			2: one dose of nivolumab as induction, then radiation (70 Gy/35 fx) and nivolumab with bolus cisplatin, then adjuvant nivolumab for seven doses		
			3: one dose of nivolumab as induction, then radiation (70 Gy/35 fx) and nivolumab with weekly cetuximab, then adjuvant nivolumab for seven doses		
			4: one dose of nivolumab as induction, then radiation (70 Gy/35 fx) with nivolumab, then adjuvant nivolumab for seven doses		
NCT03051906	DUCRO-HN: Durvalumab, Cetuximab and Radiotherapy in Head Neck Cancer	LA HNSCC	1: durvalumab every 4 weeks, cetuximab weekly, and radiation to 69.96 Gy/33 fx, followed by adjuvant durvalumab for 6 months	concurrent + sequential	1/2
NCT03247712	Neoadjuvant Immunoradiotherapy in Head & Neck Cancer	Resectable LA HNSCC	1: neoadjuvant SBRT (24–40 Gy/3–5 fx) and nivolumab, followed by surgery, followed by adjuvant nivolumab	concurrent + sequential	1/2
NCT02296684	Immunotherapy With MK-3475 in Surgically Resectable Head and Neck Squamous Cell Carcinoma	Resectable LA HNSCC, except p16-positive oropharyngeal SCC	1: two doses of pembrolizumab neoadjuvantly followed by surgery and standard risk-adapted adjuvant (chemo)radiation	sequential	2
			2: one dose of pembrolizumab neoadjuvantly, followed by surgery and standard risk-adapted adjuvant (chemo)radiation, followed by adjuvant pembrolizumab for up to six doses for patients with ENE or positive margins		
NCT03894891	Induction TPN Followed by Nivolumab With Radiation in Locoregionally Advanced Laryngeal and Hypopharyngeal Cancer	LA p16-negative SCC of larynx or hypopharynx	1: induction cisplatin, docetaxel, and nivolumab, followed by concurrent radiation and nivolumab	concurrent + sequential	2
NCT03708224	Phase II Study of Perioperative Immunotherapy in Patients With Advanced Non-Virally Associated Squamous Cell Carcinoma	Resectable LA HNSCC, except p16-positive oropharyngeal SCC	1: one dose of atezolizumab neoadjuvantly, followed by surgery and standard risk-adapted adjuvant (chemo)radiation, followed by atezolizumab every 3 weeks for up to 12 cycles	sequential	2
			2: one dose of atezolizumab and tocilizumab neoadjuvantly, followed by surgery and standard risk-adapted adjuvant (chemo)radiation, followed by atezolizumab every 3 weeks for up to 12 cycles		
NCT03426657	Radiotherapy With Double Checkpoint Blockade of Locally Advanced HNSCC	LA HNSCC	1: one cycle of induction cisplatin, docetaxel, durvalumab, and tremelimumab; patients with increased CD8+ T-cell infiltration on interval biopsy then receive durvalumab, tremelimumab, and radiation, followed by adjuvant durvalumab for 8 months	concurrent + sequential	2
NCT03532737	Concomitant Immune Check Point Inhibitor With Radiochemotherapy in Head And Neck Cancer	LA HNSCC, non-nasopharynx	1: pembrolizumab for six cycles (3 weeks each), and chemoradiation starting with cycle 2, with either bolus cisplatin or cetuximab, and radiation to 66–70 Gy/30–35 fx	concurrent + sequential	2
NCT02892201	Pembrolizumab in HNSCC With Residual Disease After Radiation	LA HNSCC with residual disease after definitive radiation	1: pembrolizumab for four cycles, followed by evaluation for salvage surgery; unresectable patients continue pembrolizumab for up to 1 year	sequential	2
NCT03721757	CA209-891: Neoadjuvant and Adjuvant Nivolumab as Immune Checkpoint Inhibition in Oral Cavity Cancer (NICO)	LA oral cavity SCC	1: one dose of neoadjuvant nivolumab followed by surgery, then one dose of nivolumab, then standard post-operative radiation or chemoradiation (60 Gy/30 fx), then 6 months of adjuvant nivolumab	sequential	2
NCT03944915	De-Escalation Therapy for Human Papillomavirus Negative Disease (DEPEND)	LA p16-negative HNSCC	1: induction carboplatin, paclitaxel, and nivolumab, followed by response-adapted chemoradiation (66–75 Gy)	sequential	2
NCT04405154	A Study of Concomitant Camrelizumab With Chemoradiation for Locally Advanced Head and Neck Cancer	LA HNSCC	1: camrelizumab for eight cycles (2 weeks each), with standard chemoradiation (bolus cisplatin and radiation [66 Gy/33 fx]) starting with cycle 2	concurrent + sequential	2
NCT02777385	Pembrolizumab in Combination With Cisplatin and Intensity Modulated Radiotherapy (IMRT) in Head and Neck Cancer	LA HNSCC, intermediate or high risk	1: pembrolizumab for one initial dose, then concurrent with radiation and weekly cisplatin, then adjuvant pembrolizumab for a total of eight doses	concurrent + sequential	2
			2: radiation and weekly cisplatin, followed by adjuvant pembrolizumab for eight doses	sequential	
NCT03383094	KEYCHAIN: Chemoradiation vs Immunotherapy and Radiation for Head and Neck Cancer	LA HNSCC, p16-positive, intermediate risk	1: pembrolizumab and standard radiation to 70 Gy/33–35 fx, followed by adjuvant pembrolizumab for up to 20 cycles (3 weeks each)	concurrent	2
			2: standard chemoradiation to 70 Gy/33–35 fx with bolus cisplatin		
NCT02707588	PembroRad: Tolerance and Efficacy of Pembrolizumab or Cetuximab Combined With RT in Patients With Locally Advanced HNSCC	LA HNSCC	1: radiation (69.96 Gy/33 fx) with concurrent pembrolizumab	concurrent	2
			2: radiation (69.96 Gy/33 fx) with concurrent cetuximab		
NCT02609503	Pembrolizumab + Radiation for Locally Adv SCC of the Head and Neck (SCCHN) Not Eligible Cisplatin	LA HNSCC, cisplatin-ineligible	1: radiation (70 Gy/35 fx) with three concurrent cycles of pembrolizumab, then three adjuvant cycles	concurrent + sequential	2
NCT03258554	NRG-HN004: Radiation Therapy With Durvalumab or Cetuximab in Treating Patients With Locoregionally Advanced Head and Neck Cancer Who Cannot Take Cisplatin	LA HNSCC, cisplatin-ineligible	1: durvalumab for seven cycles (4 weeks each); radiation to 70 Gy/35 fx starting week 2	concurrent + sequential	2/3
			2: cetuximab for eight cycles (weekly); radiation to 70 Gy/35 fx starting week 2		
NCT01810913	RTOG 1216: Testing Docetaxel-Cetuximab or the Addition of an Immunotherapy Drug, Atezolizumab, to the Usual Chemotherapy and Radiation Therapy in High-Risk Head and Neck Cancer	Resected LA HNSCC, except p16-positive oropharyngeal SCC, with pathologic ENE or positive margins	1: atezolizumab for eight cycles (3 weeks each) following surgery, with standard chemoradiation (to 60 Gy/30 fx with weekly cisplatin) starting week 2	concurrent + sequential	2/3
NCT03811015	EA3161: Testing Immunotherapy Versus Observation in Patients With HPV Throat Cancer	p16-positive oropharyngeal SCC, intermediate risk	1: radiation (70 Gy/35 fx) and concurrent weekly cisplatin, then adjuvant nivolumab for 12 months	sequential	2/3
			2: radiation (70 Gy/35 fx) and concurrent weekly cisplatin, then observation		
NCT03452137	IMvoke010: A Study of Atezolizumab (Anti−Pd-L1 Antibody) as Adjuvant Therapy After Definitive Local Therapy in Patients With High-Risk Locally Advanced Squamous Cell Carcinoma of the Head and Neck	LA HNSCC after definitive local therapy (chemoradiation or surgery + [chemo]radiation)	1: adjuvant atezolizumab for 1 year	sequential	3
			2: placebo for 1 year		
NCT03576417	NIVOPOSTOP: A Trial Evaluating the Addition of Nivolumab to Cisplatin-RT for Treatment of Cancers of the Head and Neck	Resected LA HNSCC, with ENE, positive margins, or multiple positive nodes	1: one dose of nivolumab, then nivolumab concurrent with radiation (66 Gy/33 fx) and bolus cisplatin	concurrent + sequential	3
			2: radiation (66 Gy/33 fx) with bolus cisplatin		
NCT03673735	Maintenance Immune Check-point Inhibitor Following Post-operative Chemo-radiation in Subjects With HPV-negative HNSCC (ADHERE)	Surgically resected p16-negative HNSCC with pathologic ENE or positive margins	1: one dose of induction durvalumab followed by standard chemoradiation (bolus cisplatin and radiation [66 Gy/33 fx]), followed by 6 months of adjuvant durvalumab	sequential	3
			2: standard chemoradiation (bolus cisplatin and radiation [66 Gy/33 fx])		
NCT03700905	IMSTAR-HN: Study of Nivolumab Alone or in Combination With Ipilimumab as Immunotherapy vs Standard Follow-up in Surgical Resectable HNSCC After Adjuvant Therapy	Resectable LA HNSCC, except p16-positive oropharyngeal SCC	1: one dose of neoadjuvant nivolumab followed by surgery, followed by standard risk adapted adjuvant (chemo)radiation, followed by either adjuvant nivolumab or adjuvant nivolumab+ipilimumab for 6 months	sequential	3
			2: surgical resection followed by standard risk adapted adjuvant (chemo)radiation		
NCT03765918	Study of Pembrolizumab Given Prior to Surgery and in Combination With Radiotherapy Given Post-surgery for Advanced Head and Neck Squamous Cell Carcinoma (MK-3475-689)	Resectable LA HNSCC	1: two doses of neoadjuvant pembrolizumab, then surgery, then pembrolizumab with adjuvant radiation or chemoradiation, then adjuvant pembrolizumab for 12 additional doses	concurrent + sequential	3
			2: surgery followed by adjuvant radiation or chemoradiation		
NCT03673735	ADHERE: Maintenance Immune Check-point Inhibitor Following Post-operative Chemo-radiation in Subjects With HPV-negative HNSCC	Resected LA HNSCC, except p16-positive oropharyngeal SCC, with pathologic ENE or positive margins	1: following surgery, one dose of durvalumab, then standard radiation (66 Gy/33 fx) with bolus cisplatin, then adjuvant durvalumab for six doses	sequential	3
			2: following surgery, standard radiation (66 Gy/33 fx) with bolus cisplatin		
NCT03040999	KEYNOTE-412: Study of Pembrolizumab (MK-3475) or Placebo With Chemoradiation in Participants With Locally Advanced Head and Neck Squamous Cell Carcinoma	LA HNSCC	1: one dose of induction pembrolizumab, then pembrolizumab with radiation (70 Gy/35 fx) and bolus cisplatin, then adjuvant pembrolizumab for a total of 17 doses	concurrent + sequential	3
			2: standard radiation (70 Gy/35 fx) with bolus cisplatin		
NCT02999087	REACH: Randomized Trial of Avelumab-cetuximab-radiotherapy Versus SOCs in LA SCCHN	LA HNSCC, both cisplatin eligible and ineligible	1: cetuximab and avelumab, one dose prior to radiation, then concurrent during radiation (69.96 Gy/33 fx), then adjuvant avelumab for 12 months	concurrent + sequential	3
			2: standard radiation (69.96 Gy/33 fx) with concurrent bolus cisplatin for cisplatin-eligible patients		
			3: standard radiation (69.96 Gy/33 fx) with concurrent cetuximab for cisplatin-ineligible patients		
NCT02952586	Javelin 100: Study To Compare Avelumab In Combination With Standard of Care Chemoradiotherapy (SoC CRT) Versus SoC CRT for Definitive Treatment In Patients With Locally Advanced Squamous Cell Carcinoma Of The Head And Neck	LA HNSCC	1: one dose of induction avelumab, then avelumab with radiation (70 Gy/35 fx) and bolus cisplatin, then adjuvant avelumab for 12 months	concurrent + sequential	3
			2: radiation (70 Gy/35 fx) and bolus cisplatin		

ENE, extranodal extension; fx, fraction; HNSCC, head and neck squamous cell carcinoma; ICIs, immune checkpoint inhibitors; LA, locally advanced; SBRT, stereotactic body radiotherapy.

Early results also suggest that intensification of existing chemoradiation regimens with the addition of ICIs is reasonably safe. In a small phase 1 trial of concurrent and adjuvant avelumab added to standard cetuximab/radiation in 10 cisplatin-ineligible patients with locally advanced HNSCC, no grade 4–5 toxicities were observed, and only one of eight evaluable patients discontinued avelumab for toxicity (80). REACH is a phase 3 trial that is also comparing concurrent avelumab, cetuximab, and radiation, followed by 12 months of adjuvant avelumab, against either standard bolus cisplatin with radiation or cetuximab with radiation (depending on if the patient is judged to be fit for cisplatin or not) in patients with locally advanced HNSCC; results for the 82 patients randomized during the safety phase of the trial suggested that addition of avelumab was tolerable, with 88% of patients completing concurrent avelumab as per protocol, and rates of grade 4+ events similar between control and experimental arms (81). Similarly, a single arm phase 1b study of the addition of concurrent and adjuvant pembrolizumab to standard radiation and weekly cisplatin in patients with locally advanced HNSCC demonstrated in 59 patients that concurrent pembrolizumab did not prevent patients from completing chemoradiation, and only 5 of 59 patients ultimately discontinued treatment because of irAEs (82). Finally, RTOG 3504 is a four-arm phase 1 trial in patients with intermediate or high risk HNSCC that is examining the addition of concurrent and adjuvant nivolumab to either radiation alone or radiation with weekly cisplatin, bolus cisplatin, or cetuximab; safety results from the latter three arms again demonstrated that nivolumab did not prevent timely completion of chemoradiation, and rates of dose-limiting toxicities were low (83).

Efficacy data, however, have not yet been reported from most of these or other ongoing trials. One of the single arm phase 2 trials noted above (78) of radiation with concurrent and adjuvant pembrolizumab in cisplatin-ineligible patients with locally advanced HNSCC ultimately enrolled 29 patients, and reported 1-yr progression-free survival and overall survival of 76 and 86%, respectively (84). Notably, the phase 3 Javelin 100 study is a double-blind, placebo-controlled trial that randomized 697 patients with locally advanced HNSCC to standard of care cisplatin-based chemoradiation with or without concurrent and adjuvant (for 12 months) avelumab, with progression-free survival as the primary endpoint. Unfortunately, this trial was recently terminated for likely futility after a preplanned interim analysis performed by their independent data monitoring committee (85).

Possible reasons for the failure of Javelin 100 to achieve its primary endpoint may be revealed when more complete data are available. However, in the interim, it is interesting to highlight distinctions from the successful incorporation of PD-L1 blockade into the treatment of locally advanced NSCLC as evidenced by the PACIFIC study. A predominant mode of failure in locally advanced HNSCC is locoregional recurrence (4), whereas distant metastases are more common in locally advanced NSCLC (86). Thus, examining patterns of failure in the Javelin 100 study and comparing these to patterns of failure in the PACIFIC study may inform whether ICIs in this setting are mainly eradicating systemic micrometastatic disease versus also improving local disease control. Unfortunately, PACIFIC did not collect data distinguishing intrathoracic failures within versus outside of the radiation field, highlighting the importance of thorough radiation data collection to tease out these types of questions (87). Given the high risk of lymph node metastases in patients with locally advanced HNSCC, standard radiation generally entails elective treatment of the draining cervical lymph node chains (in contrast to NSCLC, where elective lymph nodes are not intentionally irradiated). These draining lymph nodes are precisely where antigen-presenting cells migrate to for T-cell priming, following radiation to the primary tumor (21, 25). Correlative positron emission tomography–computed tomography (PET-CT) studies from a recently published clinical trial of neoadjuvant ICIs (nivolumab or nivolumab and ipilimumab) prior to surgery in patients with oral cavity SCC provides further support for the importance of the draining lymph nodes; following initiation of neoadjuvant ICIs, there was a high rate of increased fluorodeoxyglucose (FDG) uptake in the draining cervical lymph nodes on an interval PET-CT, which ultimately on surgical pathology demonstrated only reactive findings without any evidence of cancer. This observed increase in FDG uptake may therefore represent radiographic evidence of a mounting immune response (88). Given the radiosensitivity of lymphocytes, then, it seems possible that radiation (particularly longer conventionally fractionated regimens) that electively treats the draining lymph nodes following the receipt of ICI could actually hinder T-cell priming. Indeed, as noted above, there is some preclinical data to support this, as Morisada et al. demonstrated in an syngeneic mouse model of oral cavity cancer that 20 Gy in 10 fractions compared to 16 Gy in 2 fractions to both the primary tumor and the draining lymph nodes blunted tumor-specific CD8+ T-cell responses within those draining lymph nodes (although notably tumors were implanted in the mice legs and thus this is not a perfect model for head and neck lymphatics) (47). The phase 2 trial reported by Weiss et al. also noted a rate of grade 3+ lymphopenia of 58.6% (84). Another notable issue is that the design of Javelin 100, as well as many of the other trials described above, incorporated both concurrent and adjuvant ICIs in the experimental arm, whereas PACIFIC (and Checkmate-577) only tested the value of adjuvant immunotherapy. Timing and sequencing of ICIs and radiation remains a critical issue that requires further study, although the concerns regarding radiation-induced T-cell death may be particularly problematic when ICI is administered concurrently as compared with sequentially (89). Finally, as demonstrated in the preclinical work above, radiation dose and fractionation are also likely critical to successful synergy between radiation and ICIs; however, the hypofractionated regimens that appear to have the greatest immunologic potential in preclinical models differ tremendously from the long conventionally fractionated regimens (1.8–2 Gy/fraction) used in the current standard management of HNSCC. PACIFIC did also employ conventional fractionation, though standard total doses for NSCLC are somewhat lower than for HNSCC (54–66 Gy *versus* 70 Gy). Overall, given the years of experience supporting the current standard radiation regimen and fields used in the definitive management of HNSCC, careful studies will be required to determine what kinds of modifications to elective nodal irradiation, timing/sequencing, dose, and/or fractionation are required to maximize synergy with ICIs and ultimately improve patient outcomes. There is already significant heterogeneity amongst the ongoing trials in **Tables 1** and **2** with regard to these parameters, and so examining the results collectively will hopefully be informative.

## Conclusions/Future Directions

There remains excitement for the possibility of combining radiation therapy and immunotherapy to improve outcomes for patients with HNSCC. Ongoing trials will help advance this emerging field, and the developing paradigm of oligometastatic disease provides further opportunity to integrate improving systemic and local therapies. Biomarker studies conducted in parallel will also inform optimal patient selection for combined treatment approaches. Moreover, while this review has largely focused on ICIs (and PD-1/PD-L1 targeted therapies in particular) given their widespread use, immunotherapeutic agents targeting other checkpoints and pathways are in development as well (90), as are trials testing their combination with radiation (e.g. NCT04220775). Nevertheless, significant work remains to be done in both the preclinical and clinical space to determine the dose, fractionation, timing, target, and field size of radiation that will be the most synergistic with immunotherapies. Finding the optimal balance between the immunostimulatory and immunosuppressive effects of radiation is key and hopefully will herald continued improvement in outcomes for patients with HNSCC.

## Author Contributions

JQ: conceptualization; writing, original draft; writing, review and editing. JS: conceptualization; writing, review and editing. All authors contributed to the article and approved the submitted version.

## Conflict of Interest

JS reports consulting and scientific advisory board fees from Immunitas, Debiopharm, BMS, Nanobiotix, Tilos, AstraZeneca, LEK, Catenion, ACI Clinical, as well as research funding from Merck, BMS, and Regeneron.

The remaining author declares that the research was conducted in the absence of any commercial or financial relationships that could be construed as a potential conflict of interest.
